# Osteocytic Connexin43 Channels Regulate Bone–Muscle Crosstalk

**DOI:** 10.3390/cells10020237

**Published:** 2021-01-26

**Authors:** Guobin Li, Lan Zhang, Kaiting Ning, Baoqiang Yang, Francisca M. Acosta, Peng Shang, Jean X. Jiang, Huiyun Xu

**Affiliations:** 1 Key Laboratory for Space Bioscience and Biotechnology, School of Life Sciences, Northwestern Polytechnical University, Xi’an 710072, Shaanxi, China; guobinl@mail.nwpu.edu.cn (G.L.); zhanglan@mail.nwpu.edu.cn (L.Z.); ningkaiting@mail.nwpu.edu.cn (K.N.); yangbq@mail.nwpu.edu.cn (B.Y.); 2 Department of Biochemistry and Structural Biology, University of Texas Health Science Center, San Antonio, TX 78229, USA; acostafm@uthscsa.edu (F.M.A.); jiangj@uthscsa.edu (J.X.J.); 3 Key Laboratory for Space Bioscience and Biotechnology, Research & Development Institute in Shenzhen, Northwestern Polytechnical University, Shenzhen 518057, Guangdong, China; shangpeng@nwpu.edu.cn

**Keywords:** osteocytes, Cx43, gap junctions, hemichannels, bone–muscle crosstalk

## Abstract

Bone–muscle crosstalk plays an important role in skeletal biomechanical function, the progression of numerous pathological conditions, and the modulation of local and distant cellular environments. Previous work has revealed that the deletion of connexin (Cx) 43 in osteoblasts, and consequently, osteocytes, indirectly compromises skeletal muscle formation and function. However, the respective roles of Cx43-formed gap junction channels (GJs) and hemichannels (HCs) in the bone–muscle crosstalk are poorly understood. To this end, we used two Cx43 osteocyte-specific transgenic mouse models expressing dominant negative mutants, Δ130–136 (GJs and HCs functions are inhibited), and R76W (only GJs function is blocked), to determine the effect of these two types of Cx43 channels on neighboring skeletal muscle. Blockage of osteocyte Cx43 GJs and HCs in Δ130–136 mice decreased fast-twitch muscle mass with reduced muscle protein synthesis and increased muscle protein degradation. Both R76W and Δ130–136 mice exhibited decreased muscle contractile force accompanied by a fast-to-slow fiber transition in typically fast-twitch muscles. In vitro results further showed that myotube formation of C2C12 myoblasts was inhibited after treatment with the primary osteocyte conditioned media (PO CM) from R76W and Δ130–136 mice. Additionally, prostaglandin E2 (PGE2) level was significantly reduced in both the circulation and PO CM of the transgenic mice. Interestingly, the injection of PGE2 to the transgenic mice rescued fast-twitch muscle mass and function; however, this had little effect on protein synthesis and degradation. These findings indicate a channel-specific response: inhibition of osteocytic Cx43 HCs decreases fast-twitch skeletal muscle mass alongside reduced protein synthesis and increased protein degradation. In contrast, blockage of Cx43 GJs results in decreased fast-twitch skeletal muscle contractile force and myogenesis, with PGE2 partially accounting for the measured differences.

## 1. Introduction

There is an intimate relationship between bone and skeletal muscle from development, through growth, and into aging [1,2]. While such a relationship has long been considered to be primarily mechanical in nature, recently, it has been proposed that bone and muscle biochemically communicate through the actions of secreted factors [3]. On the one hand, many muscle-derived factors (termed “myokines”) such as insulin-like growth factor-1 (IGF-1), fibroblast growth factor-2 (FGF-2) [4], myostatin [5], β-aminoisobutyric acid (BAIBA) [6], and irisin [7] affect bone metabolism. On the other hand, bone cells are known to produce factors, for example, osteocalcin [8], prostaglandin E2 (PGE2) [9], Wnt3a [10], and sclerostin [11], which also influence the function of skeletal muscle. These findings suggest that bone–muscle biochemical crosstalk is essential for the optimal performance of the two tissues.

Osteocytes, which are terminally differentiated cells embedded within the bone matrix, connect to the blood vascular system through the lacunar–canalicular network [12]. It has been reported that the injection of small dyes and molecules, up to 70 kDa, to the tail vein in mice permeates the lacunar–canalicular network within a few minutes, and moreover, in humans, the number of canaliculi per osteocyte lacuna is 41 to 1 [13]. Given the high degree of vascularity of bone and the intimate connection between bone and vascular supply, it is suggested that osteocyte-specific factors could directly enter the bloodstream and target distant organs and tissues, including skeletal muscle.

Studies from Brotto’s group [9] have shown that the conditioned medium from MLO-Y4 osteocyte-like cells induces the acceleration of myogenic differentiation of C2C12 myoblasts, a phenomenon closely mimicked by the supplementation of PGE2. In addition, PGE2 signaling from osteocytes also plays an essential role in myoblast proliferation [14]. The same group later reported that the treatment of Wnt3 (a key factor secreted by osteocytes) significantly enhances myogenic differentiation [10]. Collectively, these results indicate that osteocytes (90–95% of all bone cells) and the soluble factors they produce may play a central role in bone-to-muscle crosstalk.

Osteocytes abundantly express connexin (Cx) 43 proteins, which form both gap junctions (GJs) and hemichannels (HCs). Cx43 GJs facilitate the passage of small signaling molecules (≤1.2 kDa) between coupled cells, whereas Cx43 HCs mediate communication between the cell and extracellular environment [15,16]. Given that the two types of Cx43 channels in osteocytes are portals for the passage of small molecules, and previous findings have shown that the loss of Cx43 in osteoblasts/osteocytes results in impaired muscle formation [17], this leads us to speculate that GJs and HCs play specific roles in bone–muscle crosstalk.

We have previously generated two transgenic mice driven by a 10 kb DMP1 promoter with the overexpression of Cx43 dominant negative mutants: R76W, whose GJs are inhibited while HCs are promoted; and Δ130–136, whose GJs and HCs are both blocked. Our previous studies, using these two mouse models, have shown that GJs and HCs are involved in bone remodeling [18], fracture healing [19], and bone responses to mechanical unloading [20]. In this study, we used the two transgenic mouse models to define the action of osteocytic GJs and HCs in the regulation of skeletal muscle function, which may contribute to identifying potential therapeutic approaches for pathological conditions, such as osteoporosis and sarcopenia, especially when they develop in parallel.

## 2. Materials and Methods

### 2.1. Animals

Transgenic mouse models with the overexpression of dominant negative Cx43 mutants, R76W and Δ130–136, were generated as previously described [18]. Briefly, the function of GJs was inhibited and of HCs was significantly promoted in the R76W mutant, while in the Δ130–136 mutant, both osteocytic GJs and HCs were impeded. We used 14-week-old transgenic male mice and their wild-type (WT) littermates for all biochemical analyses in this study. For PGE2 injection experiments, 1-month-old mice were treated with 80 μg/kg PGE2 (D133402, Aladdin, Shanghai, China) (dissolved in 10% sterilized saline and 90% sesame oil) or an equivalent volume of saline by intraperitoneal injection (i.p.) every other day for 4 weeks. Genotyping was performed by qPCR on genomic DNA extracted from mouse toe. The mice were maintained in a temperature-controlled room with a 12 h light/dark cycle under specific pathogen-free (SPF) conditions. Standard rodent chow and water were available ad libitum to the mice. All animal experiments were approved by the Institutional Animal Care and Use Committee (IACUC) of Northwestern Polytechnical University (NPU), Xi’an, China.

### 2.2. Muscle Mass Measurement

Animals were euthanized by cervical dislocation under anesthesia with pentobarbital sodium (45 mg/kg body weight i.p.). The gastrocnemius (GS) and soleus (SL) of both limbs were carefully dissected out and removed. Left limb muscles were weighed with electronic scales (BS224S, Sartorius, Germany) to determine differences in muscle mass. In contrast, muscles of the right limb were flash-frozen with liquid nitrogen and stored at −80 °C before being used for further biochemical analysis.

### 2.3. Histology and Immunohistochemistry

Standard hematoxylin and eosin (H&E) staining was performed on the GS and SL from transgenic and wild-type mice. Briefly, mouse muscles were dissected and fixed in 4% paraformaldehyde (P6148, Sigma, USA) in PBS (pH 7.4) for 48 h and paraffin embedded (Huayong, Shanghai, China). Five micrometer-thick cross-sections were cut from the mid-belly of each muscle. Cross-sections were deparaffinized with toluene and alcohol, followed by H&E staining.

For immunohistochemistry, post-deparaffinization, cross-sections were incubated in citric acid antigen retrieval buffer (pH 6.0) (Servicebio, Wuhan, China) for 8 min in a microwave, exposed to 3% hydrogen peroxide solution for 25 min at room temperature to quench intrinsic peroxidase activity, and then blocked in 3% bovine serum albumin (BSA; A8020, Solarbio, NanJing, China) for 30 min, prior to incubating overnight at 4 °C with a primary antibody against skeletal slow myosin (Mouse mAb, 1:2000; Sigma, USA). Tissue sections were then incubated with HRP-labeled anti-mouseIgG secondary antibody (GB23303, Servicebio) for 50 min at room temperature and developed for visualization using a diaminobenzidine-horseradish peroxidase detection system (G1211, Servicebio). Nuclei were then counterstained with hematoxylin for 3min at room temperature and mounted. All sections were photographed using an optical microscope (Model 80i, Nikon, Tokyo, Japan). Average myofiber cross-sectional area (CSA), maximal muscle CSA, myofiber CSA distribution, and fiber type composition in the entire section were analyzed by NIH ImageJ software (v1.46, Bethesda, MD, USA).

### 2.4. RNA Extraction and Real-Time PCR (qPCR)

Frozen muscles were homogenized using a tissue homogenizer (KZ-II, Servicebio) and total RNA was extracted using a Hipure Fibrous RNA Kit (R4115, Magen, Shanghai, China). Total RNA of 1 μg was used for cDNA synthesis according to the manufacturer’s protocol of HiScript^®^ II Q RT SuperMix (R223-01, Vazyme, Nanjing, China). qPCR was performed using SYBR Master Mix (R223-02, Vazyme) with a CFX96 Touch qPCR system (Bio-Rad Laboratories, Hercules, CA, USA). GAPDH was used as the housekeeping gene control and data were calculated by the 2-ΔΔCt method using the WT or WT-CM control group as the second reference and expressed as the fold change compared with the control. All specific primers (sequences see Table 1) were synthesized by Sangon Int (Shanghai, China).

### 2.5. Western Blot Analyses

GS muscle was selected for further Western blot analysis, as the decrease in muscle mass was only observed in GS but not SL. Muscle samples were lysed in ice-cold RIPA lysis buffer (P0013B, Beyotime, Haimen, China) supplemented with a protease inhibitor cocktail (1:100, CW2200S, CWBIO, Beijing, China) and a phosphatase inhibitor cocktail (1:100, CW2383S, CWBIO). The lysates were homogenized using a tissue homogenizer (KZ-II, Servicebio) and then centrifuged at 12,000× *g* for 15 min at 4 °C. The supernatants were collected, and total protein concentration was determined by a BCA Protein Assay kit (23225, Thermo Fisher Scientific). Protein samples were then mixed with 5x loading buffer and denatured at 100 °C for 5 min. An equal amount of (50 μg) total protein was subjected to SDS-PAGE using 5% stacking gel and 10% separating gel (100 V, 2 h) and then, electrically transferred (100 V, 1.5 h) onto a polyvinylidene fluoride (PVDF) membrane (IPVH00010, Merck Millipore, USA). The membranes were blocked in 5% non-fat milk powder (232100, BD Biosciences, Franklin Lakes, NJ, USA) in 1x Tris-buffered saline (TBS) with 0.1% Tween 20 (TBST) for 1.5 h at room temperature, and then incubated with primary antibodies at 4 °C overnight with the following primary antibodies: S6K1 (rabbit mAb, 1:1000; ab32359, Abcam, Cambridge, UK), p-S6K1 (Thr389, rabbit pAb, 1:250; ab32359, Abcam), 4E-BP1 (rabbit mAb, 1:1000; 9644, Cell Signaling Technology, MA, USA), p-4EBP1 (Ser65, rabbit mAb, 1:1000; 9451, Cell Signaling Technology), FoxO3a (rabbit mAb, 1:1000; 2497, Cell Signaling Technology), and Tubulin (rabbit mAb, 1:1000; ab6046, Abcam). Primary antibodies were detected with HRP-labeled anti-rabbit IgG secondary antibody (1:2000; Beyotime, Shanghai, China), which was incubated with the blots for 2 h at room temperature. After washing with TBST three times for 15 min, membranes were incubated with chemiluminescence (ECL) regents and signals were detected using a chemiluminescence detection system (T5200Multi; Shanghai, China). Quantitative analysis of band intensity was performed using NIH ImageJ software.

### 2.6. Ex vivo Muscle Force Measurement

The method of muscle force measurement was described by Ho and colleagues [21], and was performed with minor modifications. Briefly, 10 min prior to the experiment, mice were anesthetized with 5% (*v*/*v*) vaporized Isoflurane (R510-22, RWD, Shenzhen, China) mixed with O_2_. During the experiment, the mice were placed on a heated pad to maintain a physiological temperature of 30–35 °C. GS and SL muscles were dissected from surrounding tissues while maintaining its blood supply and innervation. The distal tendon of muscle was dissected and tied to a MLT0420 force transducer (Panlab, Australia) by surgical suture, while the proximal tendon of the muscle was linked to a fixed steel post. The muscles were stimulated with an electrical stimulator (SEN-3301, Tokyo, Japan) and muscle contractile force was recorded through a MLT0420 force transducer connected to a MP150 amplifier (BIOPAC, USA). Single square-wave pluses at maximal stimulation voltage (5 V) and 25 ms in duration were applied at intervals of 2 s to determine the maximal twitch force. A repeated square-wave multi-pulse at maximal stimulation voltage (5 V) and 25 ms in duration was applied at intervals of 40 ms to assess the maximal tetanic force. After force measurement, the weight and length of muscles were measured to determine muscle CSA normalized specific force [22].

### 2.7. Primary Osteocytes CM (PO CM) Preparation and Cell Culture Conditions

The isolation of primary osteocytes was performed following our published protocol with minor modifications [18]. Briefly, long bones were dissected out from our WT and two transgenic mice, and muscles were carefully removed, ensuring the periosteum was not detached. The epiphyses were cut, and bone marrow was then flushed out with sterile PBS. The bone was cut into pieces (1.5~2.0 mm in length) and digested by alternate uses of collagenase type I (BD Biosciences) and EDTA (5 mM) with constant shaking in an incubator at 37 °C and 5% CO_2_. After multiple treatments with collagenase type I and EDTA to remove other cells, such as osteoblasts and fibroblasts, the final digests were predominantly osteocytes. Isolated osteocytes were seeded on 35 mm type I collagen-coated plates and cultured in alpha Modified Eagle Medium (α-MEM, Gibco, Carlsbad, CA, USA) supplemented with 5% fetal bovine serum (FBS, Hyclone, Logan, UT, USA), 100 U/mL penicillin, and 100 μg/mL streptomycin (Beyotime, Shanghai, China). After 48 h, the CM was collected and stored at −80 °C until use.

Murine myoblasts cell line C2C12 (ATCC) cells were cultured in growth medium, which consists of high-glucose Dulbecco’s modified Eagle medium (DMEM, GIBCO, Carlsbad, CA, USA) with 10% fetal bovine serum (FBS), 100 U/mL penicillin, and 100 μg/mL streptomycin (Beyotime, Haimen, China) for 48 h, and were maintained at 50–70% cell density. Under these conditions, myoblasts proliferate but do not differentiate into myotubes. During propagation, GM was changed every 48 h. The medium was switched to differentiation medium (DM), containing 2.5% horse serum (GIBCO), 100 U/mL penicillin, and 100 μg/mL streptomycin in DMEM, to induce myogenic differentiation when cells reached 75% confluency, for select experiments, which was also changed every 48 h. All cells were maintained at 5% CO_2_ and 37 °C in a controlled humidified incubator.

### 2.8. Treatment of C2C12 Cells with PO CMs

Undifferentiated C2C12 cells were seeded in 24-well plates, 8 × 104 cells/well, and allowed to attach and grow overnight. C2C12 cells were differentiated into myotubes by replacing the GM with DM containing 25% PO CM from R76W, Δ130–136, and WT, respectively. After 48 h, medium was changed with fresh DM, and CMs were removed from the medium. Fully differentiated, functional, contractile myotubes were formed within 7 days. The cultures were fixed with 4% neutral paraformaldehyde (F1635, Sigma, USA) for 15 min and permeabilized with 0.5% Triton-X 100 (T9284, Sigma, USA) for 10 min at room temperature. Sarcomere myosin was detected with MF-20 antibody (1:100; DSHB, USA) incubated overnight at 4 °C, followed by Alexa Fluor-488-conjugated goat anti-mouse antibody (1:100; Invitrogen, USA) at room temperature for 2 h and then counterstained with DAPI. Digital images of three different regions in each replicate in three independent experiments were randomly taken after differentiation at day 7 using an inverted fluorescence microscope (Nikon 80i, Tokyo, Japan) and then, myotube area (μm2), myotube number, and fusion index (FI) were measured using ImageJ 1.46v software, where FI is defined as the ratio of the number of nuclei within myosin-expressing myotubes and total number of myogenic nuclei.

### 2.9. Intracellular Calcium (Ca^2+^) Signal Measurement

The experiment was performed following previously published protocols [23], with minor modifications. Briefly, fully differentiated myotubes were treated with 5 μM Fluo-3/AM for 30 min at 37 °C, followed by equilibration for 15 min and tested by application of 5 mM caffeine (ENZO, USA). Fluo-3/AM was excited at 488 nm, and its emission fluorescence was detected at 510 nm. Images were captured using an inverted fluorescence microscope (Nikon 80i, Tokyo, Japan) and NIH ImageJ software was used to quantify the Fluo-3 fluorescence intensity, which reveals changes in Ca^2+^ signal in myotubes. Cell imaging was recorded every 10 s within 120 s after exposure to caffeine at room temperature. The following formula was used to determine relative intracellular Ca^2+^ signal: (f_t2_−f_t1_)/f_t0_, where f_t2_ and f_t1_ are defined, respectively, as the final and initial Ca^2+^ signal every 10 s, and f_t0_ indicates Ca^2+^ signal at 0 s in each group. Three repeated experiments for each group were conducted and at least 20 myotubes for each replicate were quantified randomly for the mean Ca^2+^ signal measurement.

### 2.10. Enzyme-Linked Immunosorbent Assay (ELISA)

Blood was collected from the heart of each mouse after fasting overnight. The supernatant was obtained after centrifugation as previously described [24]. PGE2 levels in both serum and serum-free primary osteocytes CM were detected using a commercially available kit (ab133021, Abcam, USA), according to the manufacturer’s protocols.

### 2.11. Statistical Analysis

Statistical analysis was performed using Prism 6.0 software (GraphPad, La Jolla, CA, USA). All data are presented as the mean ± SD. One-way ANOVA with Tukey’s multiple comparison tests was used to compare the difference of more than two groups. A value of *p* < 0.05 was considered statistically significant for all comparisons.

## 3. Results

### 3.1. Impairment of Osteocytic HCs Reduces Fast-Twitch Muscle Mass

**Figure 1:**
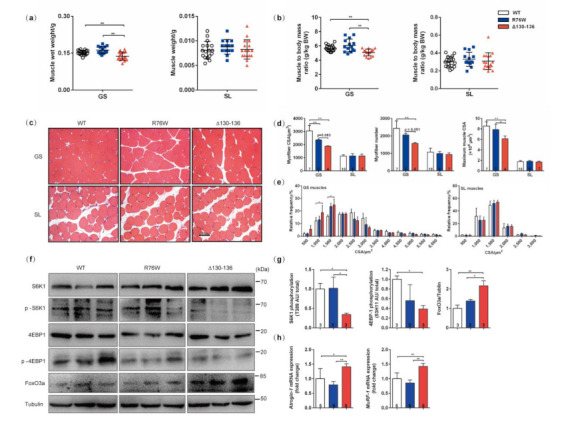
Osteocytic hemichannels (HCs) are necessary to maintain muscle mass in mice. (**a**) Muscle wet mass and (**b**) muscle to body mass ratio in transgenic mice and their WT littermates. (**c**,**d**) Entire transverse sections of the GS and SL muscles stained with H&E and measurement of myofiber number, myofiber cross-sectional area (CSA), and maximum muscle CSA. Scale bar = 50 μm. (**e**) Myofiber size distribution in GS and SL. (**f**,**g**) Phosphorylated and total S6K1 and 4ebp1 and Fox 3a protein levels in GS muscles. (**h**) The mRNA expression of MuRF-1 and Atrogin-1 in GS muscles. Sample sizes are indicated at the bottom of each column. Data are shown as mean ± SD. * *p* < 0.05; ** *p* < 0.01. GS, gastrocnemius; SL, soleus.

To investigate the effect of osteocytic Cx43 channels on muscle mass, we analyzed the wet mass of GS and SL muscles in transgenic male mice and their WT littermates. Compared with WT mice, a significant reduction in wet mass in GS (10.6%, *p* < 0.01) was observed in Δ130–136. Additionally, the wet mass of GS was 18.0% (*p* < 0.01) lower in Δ130–136 than that of R76W (Figure 1a). Similarly, the muscle to body mass ratio in GS muscle in Δ130–136 also showed a significant decrease when compared to WT and R76W, while SL was not different in either muscle wet mass or muscle to body mass ratio (Figure 1b). This decrease in muscle mass in Δ130–136 was explained, at least in part, by a decline in the myofiber cross-section area (CSA) and myofiber number (Figure 1c,d). In addition, the GS myofibers of Δ130–136 mice were small compared with those of WT littermates, as illustrated by the significant leftward shift in the distribution of myofiber CSA (Figure 1e). To test whether the decrease in muscle mass in GS is related to muscle protein turnover, the relative expression of molecules related to muscle anabolism and catabolism was determined. The results showed that the phosphorylation levels of S6K1 (p-S6K1) and 4E-BP1 (p-4E-BP1), two surrogate markers of mTOR activation, were significantly decreased in GS muscles of Δ130–136 mice compared with that of WT littermates, while there was no difference between R76W and WT (*p* < 0.05; Figure 1f,g). With regard to protein degradation, the expression of FoxO3a in GS muscle of Δ130–136 showed a significant increase compared with WT and R76W. However, there was no apparent change between WT and R76W (*p* < 0.01; Figure 1f,g). Consistent with the FoxO3a, the mRNA expression of Atrogin-1 and MuRF1 was also increased in Δ130–136 compared to WT and R76W (*p* < 0.01; Figure 1h). In all, these data suggest that the osteocyte Cx43 HCs are involved in the maintenance of muscle mass primarily in fast-twitch muscles, by affecting muscle protein synthesis and degradation. However, the impairment of Cx43 channels had little effect on muscle mass, myofiber size, and distribution in SL. Notably, GS muscle mainly comprises fast-twitch fibers, while SL muscle contains predominantly slow-twitch fibers [25,26].

### 3.2. Impairment of GJs Alters the Fiber-Type Composition and Muscle Contractile Force

**Figure 2:**
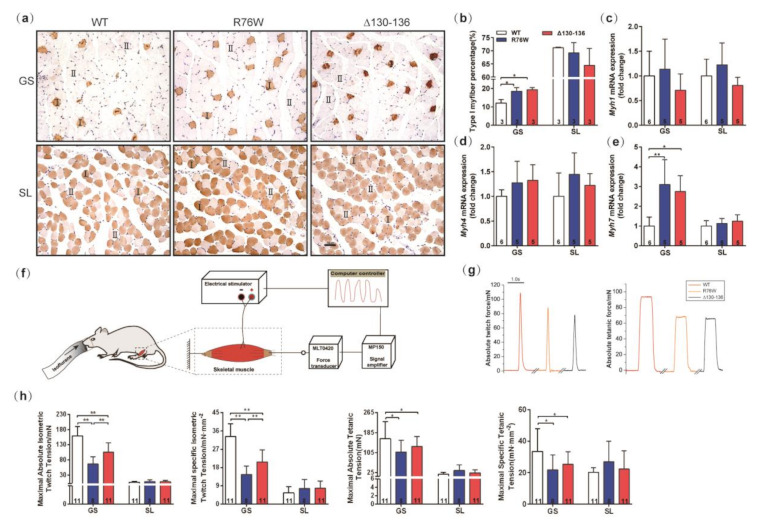
Inhibition of osteocytic GJs resulted in a shift of fast-to-slow fibers and a decrease in muscle contractile force in GS. (**a**,**b**) Representative images of myosin heavy chain type I (MyHC I) fibers and percentage of the MyHC I isoform distribution in GS and SL muscles; Scale bar = 50 μm. (**c–e**) Changes in mRNA level of Myh1 (**c**), Myh4 (**d**), and Myh7 (**e**) in GS and SL muscles, respectively. (**f**) Schematic diagram of muscle contractile force assay. (**g**) Representative twitch force and tetanic force in GS muscles. (**h**) Quantification of twitch force and tetanic force in GS and SL muscles, respectively. Sample sizes are indicated at the bottom of each column. Data are shown as mean ± SD. * *p* < 0.05; ** *p* < 0.01.

Immunohistochemistry staining showed the proportion of slow-twitch fibers (type I) in GS muscles in R76W and Δ130–136 was higher (34.9%, *p* < 0.05; and 37.7%, *p* < 0.05, respectively) than that of WT mice. However, there was no significant difference in SL muscles between transgenic and WT mice (Figure 2a,b). Within myosin heavy chain (MyHC), isoforms Myh1, Myh4, and Myh7 encode fast type IIa, IIb, and slow type I muscle fibers, respectively [26]. RT-qPCR analysis of GS muscles from transgenic mice showed that Myh7 mRNA level increased nearly threefold compared to WT, whereas the blocked Cx43 channels did not affect the mRNA expression of Myh1 and Myh4 in both GS and SL muscles (Figure 2c–e). These results indicate that inhibition of GJs in osteocytes led to the shift of fast-to-slow fibers in GS muscles.

To determine whether impaired Cx43 channels in osteocytes altered skeletal muscle function, single muscle ex vivo force tests (Figure 2f) were performed. Compared to the WT, GS muscles in Δ130–136 and R76W mice showed lower ex vivo isometric forces (Figure 2g). Specifically, the peak absolute tetanic forces of R76W and Δ130–136 were significantly reduced by 33.1% (*p* < 0.05) and 19.5% (*p* < 0.05) compared to that of WT, respectively. Moreover, compared to WT, the muscle peak specific tetanic force showed a decrease of 35.0% (*p* < 0.05) in R76W and 24.4% (*p* < 0.05) in Δ130–136, respectively. However, there was no significant difference in the SL muscles that produced a lower specific force than GS muscles (Figure 2h). This indicated that GJs in osteocytes chiefly affected fast-twitch muscle forces and fiber types.

### 3.3. Primary Osteocytes Conditioned Media (PO CM) Derived From R76W and Δ130–136 Inhibit Myogenic Differentiation of C2C12 Cells

**Figure 3:**
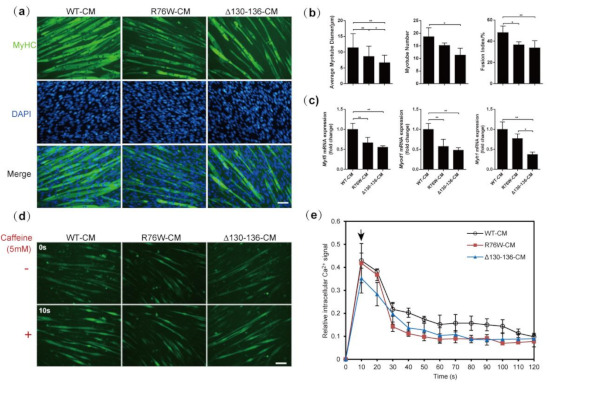
Effects of treatment with PO CMs from transgenic mice on C2C12 myoblasts myogenic differentiation. (**a**) Representative fluorescence images for sarcomere myosin (in green) and nuclei (in blue); (**b**) Quantifications of myotube diameter, myotube number, and fusion index; Scale bar = 100 μm. (**c**) mRNA expression of myogenic markers, Myf5, Myod1, and Myh1 in myotubes; (**d**,**e**) Representative fluorescence images of myotubes loaded by fluo-3/AM and quantification of Ca^2+^ signal. Arrow indicates the peak Ca^2+^ level after addition of 5 μM caffeine. Scale bar = 100 μm. *N* = 3. Data are shown as mean ± SD. * *p* < 0.05; ** *p* < 0.01.

C2C12 cells were treated with PO CM collected from the transgenic mice to determine the effect of osteocyte-derived factors on myogenic differentiation. After 7 days of differentiation, newly formed C2C12 myotubes were clearly visible in all groups (Figure 3a). Compared with WT, treatment with R76W- and Δ130–136-CM resulted in a significant decrease in average myotube diameter. Furthermore, the average myotube diameter was significantly reduced following treatment with Δ130–136-CM compared to that of R76W. Treatment with Δ130–136-CM also resulted in a significant reduction in the number and fusion index of myotubes compared to WT. R76W-CM treatment significantly reduced the fusion index of myotubes compared to that of WT (Figure 3b). RT-qPCR was also employed to determine the mRNA level of two myogenic regulatory factors (MRFs), Myf5 and Myod1, and a marker of terminal myogenic differentiation (Myh1). As expected, Δ130–136 and R76W PO CM both significantly reduced the expression of Myf5 and Myod1 in the newly formed myotubes. In addition, Myh1 also considerably decreased after Δ130–136 CM treatment compared to that of R76W and WT, but there is no apparent difference between R76W- and WT-CM treatment (Figure 3c). Both Δ130–136- and R76W-CM treatment inhibited C2C12 myotube formation, suggesting that the impairment of GJs inhibited myogenic differentiation largely affecting the secretion of soluble factors.

### 3.4. Impaired Cx43 Channels Reduces Caffeine-Induced Sarcoplasmic Reticulum Calcium Release in Myotubes

Intracellular calcium level is important for myoblast differentiation and muscle function [27,28]; 5 μM caffeine was used to effectively induce Ca^2+^ release from the sarcoplasmic reticulum of the newly formed myotubes to detect if PO CM influence cytoplasmic Ca^2+^ signal. As shown in Figure 3d,e, after 7 days of myotube differentiation, the initial Ca^2+^ signal of the response to caffeine is higher in myotubes treated with R76W- and WT-CM than that of the Δ130–136-CM group. However, the sustained Ca^2+^ signal in response to caffeine was only observed in the myotubes exposed to WT-CM over time, not in the R76W- and WT-CM groups. These results demonstrated that impaired osteocytic Cx43 channels reduced caffeine-induced Ca^2+^ release in myotubes.

### 3.5. PGE2 Treatment Augments Skeletal Muscle Mass and Contractile Force

**Figure 4:**
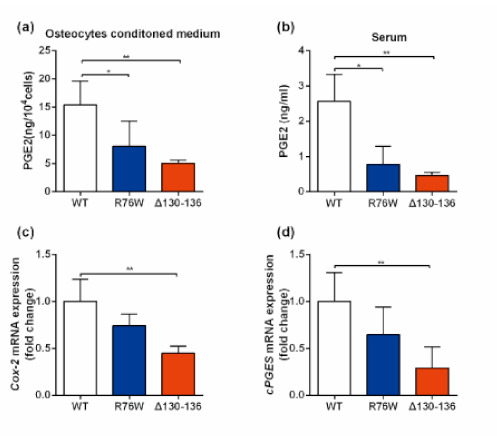
Impaired Cx43 GJs resulted to reduced PGE2 levels. (**a**,**b**) Changes in circulating PGE2 (**a**) and PGE2 content in PO CM (**b**), *N* = 3–5; (**c**,**d**) Relative expression of Cox-2 (**c**) and cPGES (**d**) in the osteocytes isolated from transgenic mice and their WT littermates. *N* = 3. Data are shown as mean ± SD. * *p* < 0.05; ** *p* < 0.01.

**Figure 5:**
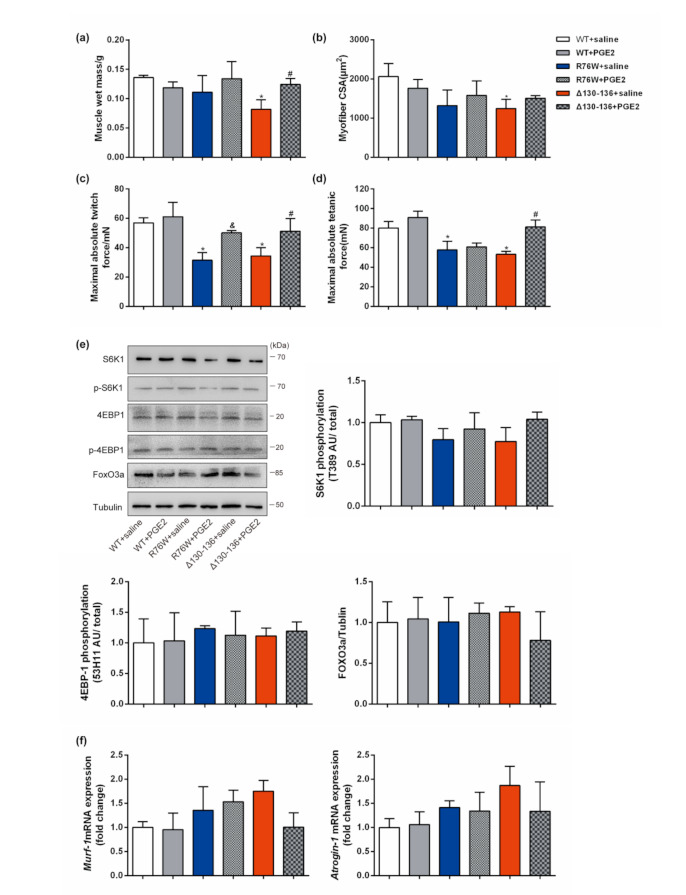
Injection of PGE2 in mice partially rescued muscle mass and contractile force. (**a**–**d**) Changes in muscle mass (**a**), average myofiber CSA (**b**), absolute muscle twitch force (**c**), and absolute muscle tetanic force (**d**) after subcutaneous injection of PGE2 in transgenic mice and WT littermates. (**e**) Western blotting analysis of p-S6K1, p-4EBP1, and Fox3a in GS muscles after PGE2 treatment in transgenic mice and WT littermates. (**f**) The mRNA expression of MuRF-1 and Atrogin-1 in GS muscles after PGE2 treatment in transgenic mice and WT littermates. *N* = 3–6/group. Data are shown as mean ± SD. * *p* < 0.05 compared to WT + saline; & *p* < 0.05 compared to R76W+saline; # *p* < 0.05 compared to Δ130–136+saline.

As shown in Figure 4a, PGE2 was detected at a lower level in PO CM from R76W and Δ130–136 compared with that of WT. Similarly, a sharp decline of circulating PGE2 was also found in R76W and Δ130–136 mice (Figure 4b). Accordingly, the mRNA expression of *Cox-2* and *cPGEs* in Δ130–136 osteocytes was significantly decreased compared to that of WT. However, no significant change was observed between R76W and WT mice (Figure 4c,d). To further confirm that these muscle phenotypes in transgenic mice are related to changes in PGE2 level, we injected PGE2 intraperitoneally to 1-month-old mice, and determined muscle mass, myofiber CSA, and muscle contractile force (absolute and specific force) in GS muscles after 4 weeks. Although PGE2 injection did not result in a full recovery of myofiber CSA (Figure 5b), the treatment did rescue GS wet mass (Figure 5a) and muscle contractile force (Figure 5c,d) in R76W and Δ130–136 mice. Interestingly, as shown in Figure 5e, the protein level of p-S6K1 and p-4EBP1 both showed a non-significant increasing trend (*p* > 0.05) in Δ130–136 mice with PGE2 treatment. In contrast, the expression of FoxO3a (Figure 5e) and *MuRF1* and *Atrogin-1* (Figure 5f) was decreased non-significantly in PGE2-treated Δ130–136 mice compared to the saline-treated control. Collectively, PGE2 was sufficient to reverse the decline in muscle mass and function due to the impairment of Cx43 HCs or GJs in transgenic mice. However, other mechanisms besides PGE2 signaling may be at work to regulate skeletal muscle protein synthesis and degradation.

## 4. Discussion

Previous studies have demonstrated that targeted deletion of Cx43 from osteoblasts in Col-Cre; Cx43-/flox mice (Cx43 cKO) leads to impaired postnatal skeletal muscle formation [17], indicating that osteoblastic Cx43 is involved in the regulation of muscle function. However, the roles of osteocytic Cx43 and Cx43 channels in bone–muscle crosstalk are still unknown. The present study, using two osteocyte-specific Cx43 transgenic mouse models, for the first time, reveals the contribution of HCs and GJs in osteocytes to the skeletal muscle.

In a previous study, Shen and colleagues report that a cKO mouse model driven by a 2.3 kb Col1α1 promoter negatively regulates the skeletal muscle mass [17]. However, they fail to distinguish the specific involvement of the GJs and HCs formed by Cx43, because Cx43 deletion ablates the function of both types of channels [29]. Our findings present that only in Δ130–136 mice, not in R76W mice, the fast-twitch muscle GS showed a decrease in muscle mass with a corresponding reduction in myofiber CSA, suggesting the impairment of osteocytic Cx43 HCs negatively regulates the skeletal muscle mass.

Muscle mass is also controlled by the balance between muscle protein synthesis and protein degradation [30]. In skeletal muscle, mTOR serves as a central regulator of protein synthesis and its activity can be assessed by determining the phosphorylation level of its downstream substrates, S6K1 and 4E-BP1 [31]. Protein degradation mainly depends upon the transcription of two major ubiquitin ligases, atrogin-1/MAFbx (Muscle Atrophy F-box) and MuRF1 (Muscle RING Finger 1), which are regulated by the transcription factor Forkhead, FoxO3a [32]. In our study, only in Δ130–136, not R76W, the phosphorylation state of both S6K1 and 4EBP1 was decreased and muscle catabolism (FoxO3a, Atrogin-1, and MuRF1) was increased. This finding was consistent with the reduced muscle mass, indicating that the impairment of Cx43 HCs reduced fast-twitch muscle mass as a result of the unbalanced muscle protein turnover with decreased protein synthesis and increased protein degradation.

Although muscle mass is known to be an essential predictor of muscle health, it is not necessarily related to muscle strength and performance. The primary function of skeletal muscle is to contract and generate force, which is closely correlated with muscle fiber types [33,34]. In this study, the impairment of osteocytic Cx43 GJs (R76W and Δ130–136) decreased in muscle-specific force. This reduction was accompanied by the increased proportion of slow-twitch type I fibers in the GS muscle, which coincides with previous reports [17]. Glycolytic type II fibers in GS, the fastest myosin isoforms, are responsible for generating force to drive locomotion, while oxidative type I fibers that are much slower than type II possess much lower contractile force and velocity but are more resistant to fatigue [26,35]. Thus, the decreased muscle contractile force in the two transgenic mice was mainly due to the shift of type II to type I fibers in GS when the function of Cx43 GJs was inhibited. On the other hand, intracellular Ca^2+^ homeostasis is a primary surrogate of skeletal muscle function [28]. Thus, the observation that decreased SR Ca^2+^ level in myotubes induced by PO CM at the vitro level could provide another explanation for the reduced muscle contractile force in transgenic mice.

Evidence from some in vitro studies indicated that osteocyte-derived factors have the potential to affect the differentiation and function of skeletal muscle cells. Treatment with CM from either MLO-Y4 or primary osteocytes promoted C2C12 myogenic differentiation [9,10]. In contrast, the differentiation was significantly inhibited by the secretome of mechanically activated Ocy454 osteocytic cells, possibly due to the difference in cell lineages and experimental conditions [36]. We showed here that treatment with PO CM from both Δ130–136 and R76W mice significantly inhibited the differentiation of myoblasts into myotubes, as evidenced by the decrease in fusion index, down-regulation of myogenic regulatory factors (*Myf5* and *Myod1*), and reduction in caffeine-induced SR calcium release. Given that the Δ130–136 mutant has a dominant negative effect on both HCs and GJs, while R76W only on GJs, these in vitro results indicated that inhibition of Cx43 GJs was correlated with impaired myogenesis.

These findings proved that osteocytes could release factors through Cx43-formed channels that, in turn, affect skeletal muscle function. We have previously reported that PGE_2_, released by Cx43 HCs in response to mechanical strain [37], is also critical for GJ-mediated intercellular communication [38], indicating that alteration in the function of the two channels may directly affect the diffusion of PGE_2_. In this study, we found that a relatively low level of PGE_2_ was detected in both the two Cx43 mutants (at the in vitro and in vivo level). This finding may be explained by the fact that the inhibition of GJs neutralized the facilitating effect of Cx43 HCs on PGE_2_. Additionally, Cx43 GJs have been shown to affect cellular function through regulating osteogenic gene transcription [39]. Thus, the disruption of Cx43 GJs in osteocytes resulted in down-regulation of the expression of COX-2 and cPGEs, two forms of PGE_2_ synthase [21], which could be another plausible explanation for the lower amount of PGE_2_ in Δ130–136. However, this was in contrast to the work by Shen et al. [17], who reported that plasma prostaglandin E metabolites (PGEM) were markedly increased in Cx43 cKO mice compared to WT mice. This discrepancy between the two mouse models may be caused by the difference in the temporal and spatial expression of Cx43. Col-Cre cKO mice lack Cx43 in osteoblasts and osteocytes [17,40], while our transgenic mouse model supports stable Cx43 expression despite impaired channel function [20]. In addition, the channel-independent functions of Cx43, such as interaction with other transcription factors to regulate gene expression, could also be responsible for the variable results [41].

The association between alterations in PGE_2_ level and skeletal muscle phenotypes in the two mutants was further supported by the results from our in vivo rescue experiment, which showed that PGE_2_ injection into the transgenic mice increased muscle mass and function. However, our results also showed PGE_2_ treatment has a tendency of increased muscle protein synthesis and decreased protein degradation, but no significant difference was observed between PGE_2_-treated Δ130–136 mice and those treated with saline. This finding is similar to a previous study showing that PGE_2_ had little or no effect on the regulation of muscle protein synthesis and protein degradation [42] in isolated muscles both in mice [43] and rat [44]. Based on these results, it appears that the regulation of muscle mass by PGE_2_ is not fully caused by the alteration in muscle protein turnover. That being said, how other osteocyte-derived factors contribute to the regulation of muscle protein synthesis and degradation is not explored in this study; this is the subject of our continuing studies.

## 5. Conclusions

**Figure 6:**
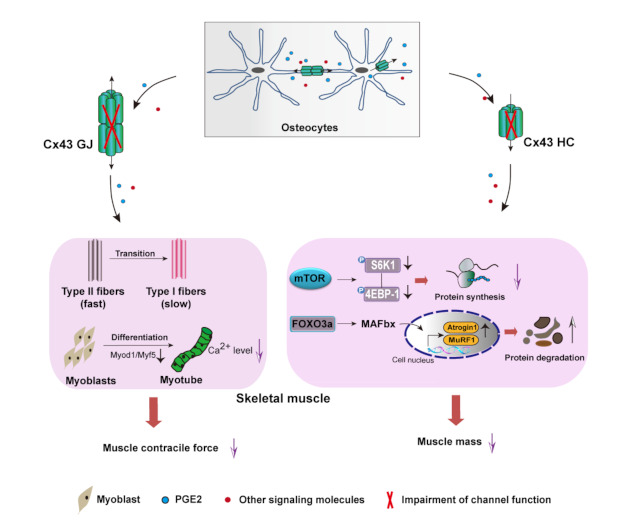
Proposal model of the role of Cx43 HCs and GJs in bone–muscle crosstalk. Cx43-formed HCs and GJs in osteocytes are important portals for the cell-to-cell and cell-to-extracellular environment communication, respectively. Impairment of HCs resulted in decreased fast-twitch muscle mass with reduced protein synthesis and increased protein degradation, whereas impairment of GJs inhibited C2C12 myogenic differentiation and decreased fast-twitch muscle contractile force accompanied by the shift of fast-to-slow fibers. PGE2 could effectively rescue the decline in muscle mass and contractile force independently of muscle protein turnover, thereby supporting a more complex mechanism in bone–muscle crosstalk that requires the coordinated participation of multiple factors.

This study indicates that Cx43 channels are involved in bone–muscle crosstalk and PGE_2_ is one of the potent factors in this regulation. Specifically, impairment of Cx43 HCs decreases the fast-twitch muscle mass with reduced protein synthesis and increased protein degradation, while inhibition of GJs reduces muscle contractile force, myogenic differentiation, and regulates fast-to-slow fiber transition. Among the factors produced by osteocytes, PGE_2_ could positively regulate fast-twitch muscle mass independent of muscle protein turnover (Figure 6). Such knowledge has profound implications for identifying potential novel target therapies for diseases where bone–muscle crosstalk is key, such as osteoporosis and sarcopenia, especially when they co-exist.

## Figures and Tables

**Table 1:** Primer sequences for qPCR.

Gene Name	Primer Sequences (5′ to 3′)
*mCx43*	Forward: CGGAAGCACCATCTCCAACT
	Reverse: CCACGATAGCTAAGGGCTGG
*mCx50*	Forward: CAAGGGCTGTCTGCTGAGAA
	Reverse: AGATCATCTGACCTGGCCCT
*Myh1*	Forward: GCTTCAAGTTTGGACCCACG
	Reverse: TTCTGAGCCTCGATTCGCTC
*Myh4*	Forward: GGAGGCTGAGGAACAATCCA
	Reverse: TCTCCTGTCACCTCTCAACAG
*Myh7*	Forward: CAACCTGTCCAAGTTCCGCA
	Reverse: TACTCCTCATTCAGGCCCTTG
*Myod1*	Forward: TACGACACCGCCTACTACAGTG
	Reverse: GTGGTGCATCTGCCAAAAG
*Myf5*	Forward: CTGTCTGGTCCCGAAAGAAC
	Reverse: TGGAGAGAGGGAAGCTGTGT
*Cox-2*	Forward: TGAGCAACTATTCCAAACCAGC
	Reverse: GCACGTAGTCTTCGATCACTATC
*cPGEs*	Forward: AAGGAGAATCTGGCCAGTCA
	Reverse: ATCCTCATCACCACCCATGT
*MuRF1*	Forward: GCCATCCTGGACGAGAAGAA
	Reverse: CAGCTGGCAGCCCTTGGA
*Atrogin-1*	Forward: AGACCGGCTACTGTGGAAGAG
	Reverse: CCGTGCATGGATGGTCAGTG
*GAPDH*	Forward: TCAACAGCAACTCCCACTCTTCCA
	Reverse: ACCCTGTTGCTGTAGCCGTATTCA

## Data Availability

Data are contained within the article.
